# RETRACTED: Asweto et al. Combined Effect of Silica Nanoparticles and Benzo[a]pyrene on Cell Cycle Arrest Induction and Apoptosis in Human Umbilical Vein Endothelial Cells. *Int. J. Environ. Res. Public Health* 2017, *14*, 289

**DOI:** 10.3390/ijerph22040508

**Published:** 2025-03-27

**Authors:** Collins Otieno Asweto, Jing Wu, Hejing Hu, Lin Feng, Xiaozhe Yang, Junchao Duan, Zhiwei Sun

**Affiliations:** 1 Department of Toxicology and Sanitary Chemistry, School of Public Health, Capital Medical University, Beijing 100069, China; asweto_collins@yahoo.com (C.O.A.); wujing000032@126.com (J.W.); jingjinghe_2008@126.com (H.H.); fenglin615@163.com (L.F.); yangxiaozhe3@163.com (X.Y.); 2 Beijing Key Laboratory of Environmental Toxicology, Capital Medical University, Beijing 100069, China

The journal retracts the article titled “Combined Effect of Silica Nanoparticles and Benzo[a]pyrene on Cell Cycle Arrest Induction and Apoptosis in Human Umbilical Vein Endothelial Cells” [1].

Following its publication, concerns were brought to the attention of the Editorial Office regarding image irregularities contained within the article [1].

Adhering to our complaint procedure, the Editorial Office and Editorial Board conducted an investigation that confirmed a duplication of images B and D in Figure 8, presenting different experimental conditions and a partial overlap between Figure 6 in the publication [1] and Figure 5 from another publication by a different authorship group [2]. Therefore, the Editorial Board and the authors have decided to retract this publication [1], as per MDPI’s retraction policy (https://www.mdpi.com/ethics#_bookmark30).

This retraction was approved by the Editor-in-Chief of the journal *IJERPH*.

Junchao Duan and Zhiwei Sun agree to this retraction. The remaining authors did not provide a comment on this decision.
